# Hypoxia and the Anticoagulants Dalteparin and Acetylsalicylic Acid Affect Human Placental Amino Acid Transport

**DOI:** 10.1371/journal.pone.0099217

**Published:** 2014-06-05

**Authors:** Marc-Jens Kleppa, Sarah-Vanessa Erlenwein, Natallia Darashchonak, Constantin S. von Kaisenberg, Frauke von Versen-Höynck

**Affiliations:** Department of Obstetrics and Gynecology, Hannover Medical School, Hannover, Germany; Medical Faculty, Otto-von-Guericke University Magdeburg, Medical Faculty, Germany

## Abstract

**Background:**

Anticoagulants, e.g. low-molecular weight heparins (LMWHs) and acetylsalicylic acid (ASA) are prescribed to women at risk for pregnancy complications that are associated with impaired placentation and placental hypoxia. Beyond their role as anticoagulants these compounds exhibit direct effects on trophoblast but their impact on placental function is unknown. The amino acid transport systems A and L, which preferably transfer essential amino acids, are well-described models to study placental nutrient transport. We aimed to examine the effect of hypoxia, LMWHs and ASA on the activity of the placental amino acid transport systems A and L and associated signalling mechanisms.

**Methods:**

The uptake of C^14^-MeAIB (system A) or H^3^-leucin (system L) was investigated after incubation of primary villous fragments isolated from term placentas. Villous tissue was incubated at 2% O_2_ (hypoxia), 8% O_2_ and standard culture conditions (21% O_2_) or at 2% O_2_ and 21% O_2_ with dalteparin or ASA. Activation of the JAK/STAT or mTOR signalling pathways was determined by Western analysis of total and phosphorylated STAT3 or Raptor.

**Results:**

Hypoxia decreased system A mediated MeAIB uptake and increased system L mediated leucine uptake compared to standard culture conditions (21% O_2_). This was accompanied by an impairment of STAT3 and a stimulation of Raptor signalling. System L activity increased at 8% O_2_. Dalteparin treatment reduced system A and system L activity under normoxic conditions and ASA (1 mM) decreased system A and L transporter activity under normoxic and hypoxic conditions.

**Conclusions:**

Our data underline the dependency of placental function on oxygen supply. LMWHs and ASA are not able to reverse the effects of hypoxia on placental amino acid transport. These findings and the uncovering of the signalling mechanisms in more detail will help to understand the impact of LMWHs and ASA on placental function and fetal growth.

## Introduction

Adequate placental development during pregnancy is a major determinant of pregnancy outcome. Several changes in placental morphology and function have been described in pregnancies complicated by preeclampsia, fetal intrauterine growth restriction (IUGR), placental infarction and recurrent abortions [1]. Among these alterations, impaired invasion of the extravillous trophoblast and failed vascular remodeling of the maternal spiral arteries is thought to result in impaired uterine blood flow to the developing placenta, contributing to intermittent hypoxia and placental dysfunction [2], [3], [4].

Anticoagulants, e.g. low-molecular weight heparins (LMWHs) and low-dose acetylsalicylic acid (ASA), are widely used in obstetric practice to improve pregnancy outcome in women at risk for the above-mentioned complications [5], [6], [7]. Beyond the classical anticoagulant role LMWHs and ASA exert a variety of non-anticoagulant actions including direct effects on the trophoblast. Heparin is involved in the regulation of differentiation and proliferation of villous cytotrophoblasts [8], [9], [10] and the anti-angiogenic factor soluble fms-like tyrosine kinase-1 (sFLT1) [11], [12].

The placenta plays a central role in nutrient transport between the maternal and fetal compartments. Amino acids are important nutrients and normal amino acid transport from mother to fetus is essential for adequate fetal growth. System A and L are two amino acid transport systems that have been well characterized during the last decades. The system A amino acid transport system is the most widely studied placental amino acid transporter and a ubiquitous sodium dependent system that actively transports small, zwitterionic, neutral amino acids with short unbranched side chains such as alanine, serine, and glutamine [13]. The system L transporter, a sodium independent transporter, is involved in the transport of branched chain essential amino acids, e.g leucine and phenylalanine [14]. The mTOR and JAK/STAT signalling pathways play a central role in the regulation of placental amino acid transporter activity and are modulated in pregnancy complications associated with altered fetal growth [15], [16], [17]. Decreased placental system A activity accompanied by changes in the mTOR signalling cascade have been illustrated in pregnancies complicated by IUGR, and increased activities in diabetic pregnancies contribute to altered fetal growth patterns [18], [19]. One of the few studies available examining the effect of anticoagulants on amino acid transport reported a decrease in the transport of the amino acid histidine has been reported after ASA treatment of rat intestine [20].

We tested the hypothesis that placental system A and L activity are affected by hypoxic oxygen conditions and that LMWHs or ASA interact with placental villi in a non-anticoagulant manner to affect placental amino acid transport.

## Materials and Methods

### Chemicals and Buffers

All experiments using placental tissue were carried out using Tyrode’s buffer (consisting of 135 mM NaCl or 135 nM choline chloride for sodium-free Tyrode’s buffer, 5 mM KCL, 1.8 mM CaCl_2_, 1 mM MgCl_2_, HEPES 10 mM and 5.6 mM dextrose). The pH was adjusted with NaOH (sodium containing buffer) or KOH (sodium free buffer) to pH 7.4. Placental tissues were incubated and dissected in a buffer consisting of 1 volume of Dulbecco’s Modified Eagle’s medium (DMEM, containing 5.6 mM glucose, amino acids, vitamins, minerals) mixed with 3 volumes of sodium containing Tyrode’s buffer (DMEM/Tyrode’s, 1∶3 vol/vol) to produce a nearly physiological concentration of amino acids.

[C14]-Methylaminoisobutyric acid (C^14^-MeAIB, Hartmann Analytics, Braunschweig, Germany, 0.1 mCi/ml; specific activity: 55 mCi/mmol), a radiolabeled non-metabolizable amino acid analog with system A specificity was added to Tyrode’s buffer (with and without sodium) in the presence or absence of the below listed experimental treatments to achieve a final concentration of 1.7 µmol/l [C^14^]-MeAIB. System L activity was measured by determining uptake of [H^3^]-leucine (Hartmann Analytics, Braunschweig, Germany, 1 mCi/ml; specific activity 140 mCi/mmol) in the presence and absence of 1 mM 2-amino-2-norbornanecarboxylic acid (BCH, Sigma-Aldrich Chemie GmbH, Taufkirchen, Germany), which specifically blocks system L activity, at a final concentration of 0.005 µmol/l [H^3^]-leucine.

Protein extraction buffer consisted of 50 mM Tris (pH 7.4), 150 mM NaCl, 2 mM EGTA, 2 mM EDTA, 25 mM NaF, 25 mM β-glycerolphosphat, 0.1 mM Na-V, 0.1 mM PMSF, leupeptin, aprotinine, 0.2% Triton X-100 and 0.3% NP-40.

All primary antibodies were purchased from New England Biolabs GmbH (Frankfurt, Germany), the secondary antibodies from SIGMA Aldrich (Taufkirchen, Germany).

### Tissue Collection and Preparation of Villous Explants

The Ethical Committee at Hannover Medical School approved this study (No. 3254) and written informed consent was obtained from all subjects. Fresh placental tissue was obtained immediately after a singleton delivery from healthy women who were giving birth to full-term infants in the absence of labour by caesarean section at Hannover Medical School. Decidua was removed from placenta and biopsies were taken from the maternal side between the peripheral edge of the placenta that was free of infarcts and the insertion of the umbilical cord. The biopsies were directly transported to the laboratory in DMEM/Tyrodes buffer, which was pre-equilibrated at 8% O_2_. Placental tissue was washed several times to remove blood and dissected into small villous fragments (2 mm^3^) in DMEM/Tyrode’s buffer. Villous tissue (0.5–1.5 mg protein/well) was put on netwell inserts and placed in the appropriate well of a 12-well cell culture plate containing the prepared treatment solutions. All dissection procedures were performed at 37°C and at an oxygen level of 8% O_2_. Previous validation experiments for transport studies demonstrated maintained functional and structural integrity of villous fragments for at least 4 h in explant culture [21], [22].

### Measurement of System A and L Transport Activity

The effect of different oxygen conditions (2%, 8% and 21% O_2_), the low molecular weight heparin dalteparin and acetylsalicylic acid (ASA) on system A and L activity were studied. Villous tissues were preincubated for 2 h in 2 ml of DMEM/Tyrode's buffer in the presence or absence of ASA (Sigma-Aldrich Chemie GmbH, Taufkirchen, Germany) at 0.01 mM (∼75 mg/d), 0.1 mM (∼125 mg/d) and 1 mM (∼150 mg/d) [23] or dalteparin (Fragmin P, Pfizer Pharma GmbH, Berlin, Germany) of the same lot in concentrations of 0.025 IU/ml (∼2500 IU/d), 0.25 IU/ml (∼5000 IU/d) and 2.5 IU/ml (∼10.000 IU/d) [24] at 37°C under continuous gently shaking. Concentration ranges of dalteparin and ASA were chosen according to previous studies [25], [26].

After preincubation villous tissue was washed three times with 2 ml of sodium containing or sodium free Tyrode’s buffer with 1 mM BCH at 37°C. Afterwards the explants were incubated for 20 min at 37°C in 1.75 ml Tyrode’s buffer containing [C^14^]-MeAIB (1.7 µmol/L) and [H^3^]-leucin (0.005 µmol/L) with or without sodium under constant agitation. The uptake of [C^14^]-MeAIB and [H^3^]-leucin was stopped by washing the tissue 4 times in ice-cooled sodium free Tyrode’s buffer for 2 min each by swirling. The tissue fragments were placed in 2 ml of distilled water overnight to release the accumulated [C^14^]-MeAIB and [H^3^]-leucin. The following day the protein concentration of villous tissue was determined according to the method of Bradford using a protein assay procedure (Bio-Rad Laboratories GmbH, Munich, Germany) and bovine serum albumin (BSA) as the standard. After villous tissue was removed 1 ml liquid scintillation fluid was added to 1 ml of aqueous supernatant, vortexed and the radioactivity measured by a scintillation counter.

In all uptake experiments, each experimental condition was studied in triplicate. The uptakes of [C^14^]-MeAIB and [H^3^]-leucin were calculated by subtracting non-mediated uptake (sodium-free Tyrode’s solution for system A and Tyrode’s solution containing 1 mM BCH for system L) from the uptake in the buffer representing total uptake (sodium containing buffer). After normalizing to the total protein amount of its respective fragment the activity was expressed as pmol of [C^14^]-MeAIB uptake per mg of protein per 20 min or as nmol of [H^3^]-leucin uptake per mg of protein per 20 min.

### Tissue Viability and hCG Determination

Briefly, we assayed for lactate dehydrogenase (In vitro toxicology LDH assay kit, Sigma–Aldrich Chemie GmbH, Taufkirchen, Germany) and hCG release into the media after a 2 h incubation. After placental preparation the control was obtained by sonicating fresh placental villous explants (equivalent to the amount of explants used per well) from the same placentae in the same culture media used for experimentation. LDH values were quantified as a proportion of the 100% LDH control. Quantitative detection of hCG was performed with a solid-phase, two-step chemiluminescent immunometric assay (Immulite/Immulite 1000 analyzer, Siemens).

### Western Blot Analysis

Western blot analysis was performed according to published protocols [13]. The membrane was incubated at 4°C overnight with specific antibodies directed against STAT3 (1∶1000), pSTAT3 (1∶2000 diluted in distilled water with 5% BSA), Raptor (1∶500), p-Raptor (1∶500) or β-actin (1∶3000). After incubation with primary antibodies, the membrane was washed three times in TBS-0.1% Tween 20 for 15 min each before incubated with species specific secondary antibodies (1∶10000) for 1 h at room temperature. Secondary antibodies were diluted in TBS-0.1% Tween 20 with 5% milk if not differently indicated. To visualize the bands the membrane was incubated in clarity western ECL substrate (Thermo Fisher Scientific, Bonn, Germany) for 5 min for chemiluminescent detection and exposed to an ECL hyperfilm (Th. Geyer GmbH & Co. KG, Hamburg, Germany). For the analysis of β-actin, the membranes were stripped and reprobed with an anti β-actin antibody to account for protein loading variations. Relative density of bands was evaluated by densitometry with Image J software. The mean density of the untreated sample ( =  control) bands was assigned an arbitrary value of 1, and the mean density of the treated groups are expressed relative to the control groups.

### Data Presentation and Statistical Analysis

The number of experiments (*n*) represents the number of different placentas studied. In the amino acid uptake studies each condition was studied in triplicates. Data are normalized in individual experiments relative to the internal control treatment to account for inter-tissue variation of system A and L activity or protein expression. Data are presented as mean and standard error (SEM). Protein phosphorylation data are expressed as the pSTAT3/STAT3 or pRaptor/Raptor ratio after adjusting for β-actin [13]. After testing for normality distribution by Shapiro Wilk or Kolmogorov Smirnov test differences between control and experimental groups were analyzed with unpaired t test. Test for linear trend was used to analyze a concentration-depending effect of ASA or dalteparin on system A or L transporter activity or on protein expression. Statistical analyses were performed using GraphPad software (Version 4.03). P values <0.05 were considered significant. Table 1 represents an overview of the significant results.

**Table 1:** The table summarizes the different effects of dalteparin, ASA and hypoxia on system A and L amino acid transport, pSTAT3/STAT3 and pRaptor/Raptor ratio.

		system A activity	system L activity	pSTAT3/STAT3	pRaptor/Raptor
hypoxia	8% O_2_	–	76% ↑	–	–
	2% O_2_	27% ↓	42% ↑	32% ↓	36% ↑
Dalteparin 21% O_2_	0.025 IU/ml	17% ↓	22% ↓	–	–
	0.25 IU/ml	21% ↓	31% ↓	–	48% ↑
	2.5 IU/ml	22% ↓	24% ↓	–	–
Dalteparin 2% O_2_	0.025 IU/ml		–	–	–
	0.25 IU/ml		–	–	–
	2.5 IU/ml		–	42% ↓	–
ASA 21% O_2_	0.01 mM	–	–	–	34% ↓
	0.1 mM	–	–	–	25% ↓
	1 mM	22% ↓	–	–	–
ASA 2% O_2_	0.01 mM	–	29% ↓	–	–
	0.1 mM	–	–	–	–
	1 mM	31% ↓	21% ↓	–	–

↑ = increase; ↓ = decrease; – = no significant change.

## Results

### Hypoxia Inhibits Placental System A and Stimulates System L Transporter Activity

**Figure 1:**
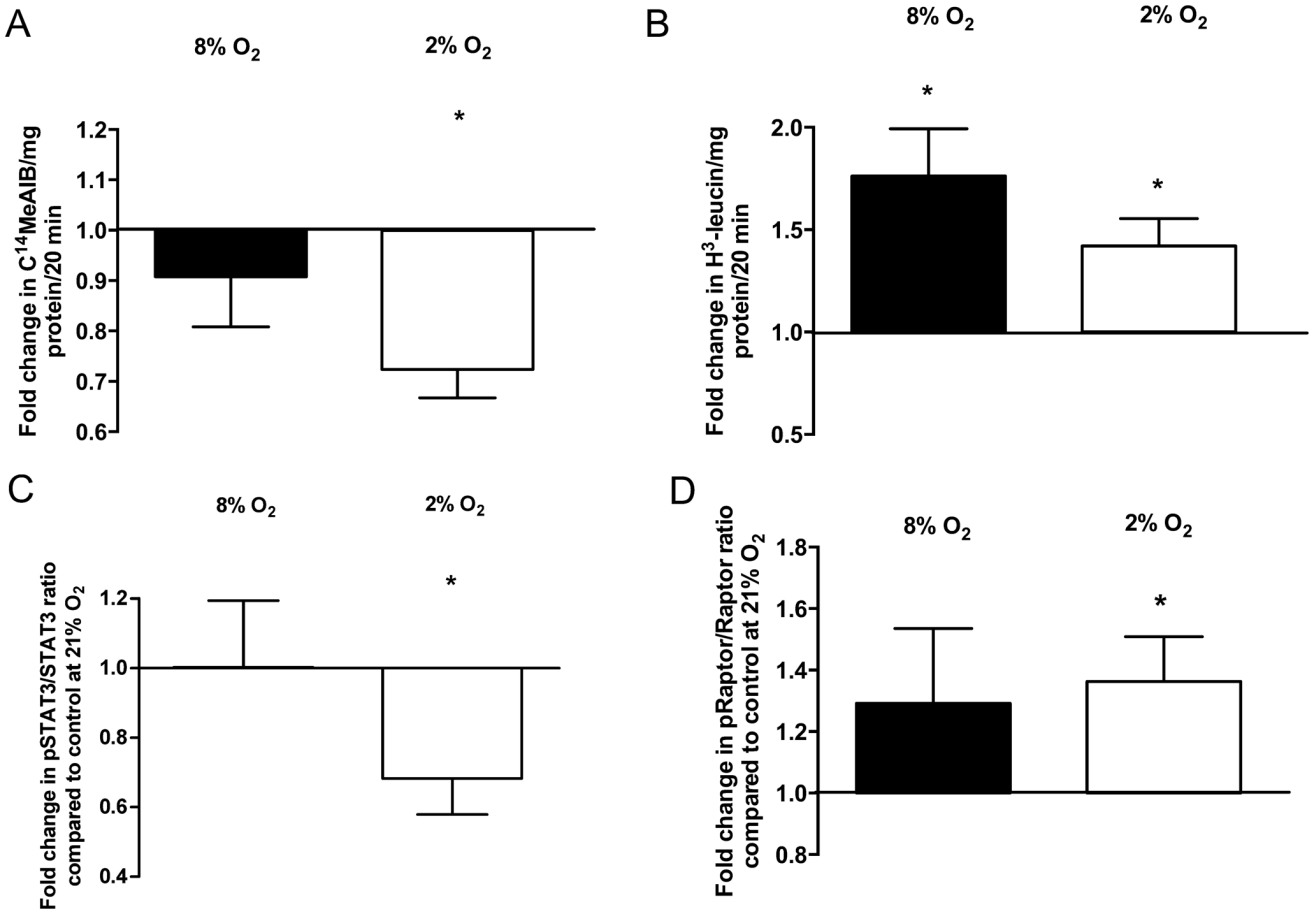
Relative placental system A (A) and L amino acid transporter activities (B) after 2 h of incubation of villous explants at 2% O_2_ or 8% O_2_ compared to 21% O_2_. Normalized ratio of pSTAT3/STAT3 (C) and pRaptor/Raptor (D) at different oxygen concentrations. Values are relative means ± SEM. *p<0.05 compared to system A and L activities or ratio of pSTAT3/STAT3 and pRaptor/Raptor incubated at 21% O_2_ (set to 1).

Hypoxic culture conditions (2% O_2_) decreased system A amino acid transporter activity by 27% in placental villous explants after 2 h compared to standard culture conditions (21% O_2_), (0.73±0.07, p<0.001, n = 22, figure 1A). In contrast to system A, the transporter activity of system L significantly increased by 42% under hypoxic conditions compared to an oxygen level of 21% O_2_ (1.42±0.13, p = 0.01, n = 22, figure 1B). At 8% O_2_ the system A transporter activity was not significantly different compared to 21% O_2_ (0.91±0.12, p = 0.47, n = 9, figure 1A), but system L amino acid transport activity increased by 76% (1.76±0.24, p = 0.01, n = 8, figure 1B, table 1).

### Hypoxia Decreases Phosphorylation of STAT3 and Increases Phosphorylation of mTOR

To study the effect of hypoxia on the signalling pathways involved in placental system A and L amino acid transport we next determined the expression of STAT3 and Raptor and their phosphorylated forms. While hypoxia decreased STAT3 phosphorylation (pSTAT3/STAT3∶0.68±0.1, p = 0.009, n = 13, figure 1C) the phosphorylation of Raptor was increased significantly by 36% under hypoxic conditions compared to 21% O_2_ (pRaptor/Raptor: 1.36±0.15, p = 0.03, n = 16, figure 1D, table 1). Phosphorylation of STAT3 (1.0±0.19, n = 5, p = 0.99) and Raptor (1.29±0.28, p = 0.29, n = 5) did not change significantly at 8% O_2_ compared to standard culture conditions.

### Dalteparin and Acetylsalicylic Acid Decrease Placental System A and System L Transporter Activities under Standard Culture Conditions

**Figure 2:**
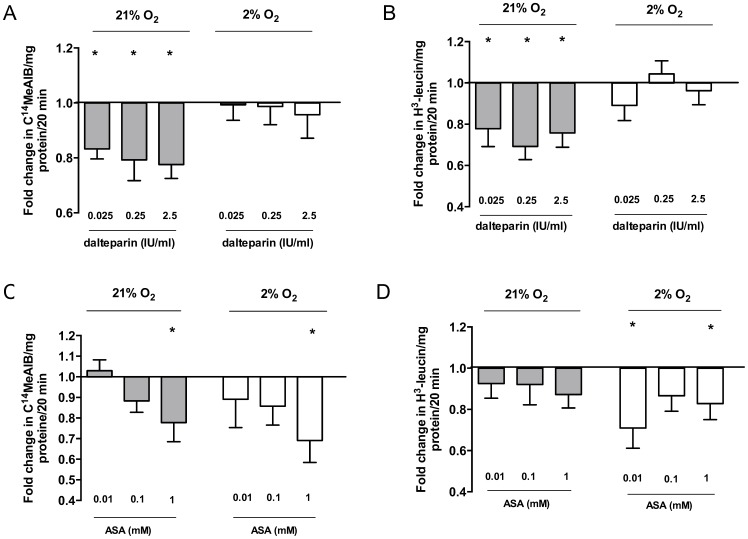
System A (A, C) and L (B, D) amino acid transporter activities in placental villous explants after 2 h of incubation with dalteparin (0.025 IU/ml, 0.25 IU/ml, 2.5 IU/ml) or with ASA (0.01 mM, 0.1 mM, 1 mM) at 21% and 2% O_2_. Values are relative means ± SEM. *p<0.05 compared to untreated control (set to 1).

To examine the effect of anticoagulants we performed amino acid transport assays in the presence of increasing concentrations of dalteparin or ASA. Dalteparin decreased system A amino acid transport activity of placental villous explants at 21% O_2_ and at a concentration of 0.025 IU/ml (0.83±0.04, p = 0.001), 0.25 IU/ml (0.79±0.07, p = 0.03) and 2.5 IU/ml (0.78±0.05, p = 0.001, table 1) compared to untreated villous explants (n = 6, figure 2A).

System L amino acid transporter activity was also reduced under standard culture conditions by 22%, 31% and 24% at 0.025 IU/ml (0.78±0.08, p = 0.04), 0.25 IU/ml (0.69±0.06, p = 0.003) and 2.5 IU/ml dalteparin (0.76±0.07, p = 0.006, table 1) compared to control (n = 6–7, figure 2B). There was no significant effect of dalteparin on system A and L amino acid transport activity under hypoxic conditions (n = 7).

System A amino acid transport activity was impaired by 22% and 31% in villous explants treated with 1 mM ASA compared to untreated control at 21% O_2_ (0.78±0.13, p = 0.03) and 2% O_2_ (0.69±0.11, p = 0.02), respectively (figure 2C), (n = 15 and 9). System L amino acid transporter activity was reduced by 29% and 21% after treatment with 0.01 mM (0.71±0.1, p = 0.03, n = 10, table 1) and 1 mM ASA (0.83±0.08, p = 0.04, n = 10, table 1) at 2% O_2_ but not significantly at 0.1 mM ASA (0.87±0.08, p = 0.2, n = 10, figure 2D). System L transport did not change under standard culture conditions. There was a concentration dependent effect of ASA treatment on system A amino acid transporter activity at 21% (p = 0.01).

### Effect of Dalteparin on Phosphorylation of STAT3 and Raptor at Different Oxygen Levels

**Figure 3:**
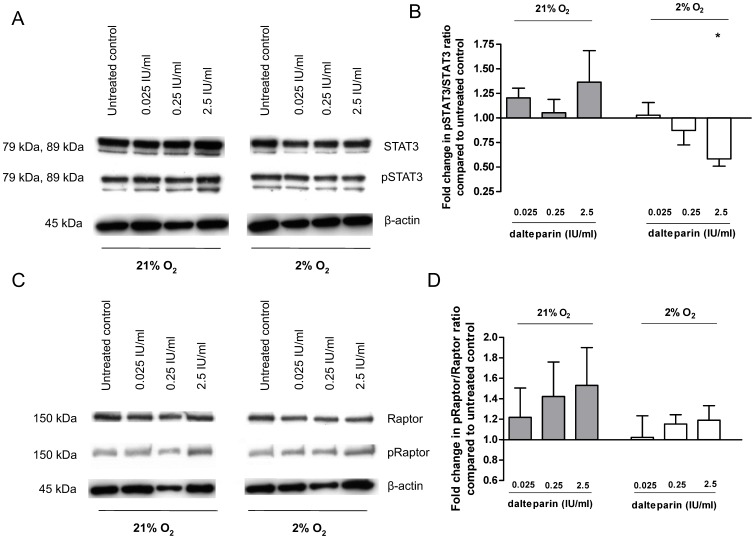
Protein expression of pSTAT3/STAT3 (A, B) and pRaptor/Raptor (C, D) in placental villous explants after incubation with dalteparin (0.025 IU/ml, 0.25 IU/ml, 2.5 IU/ml) at 21% and 2% O_2_. Representative Western blot of STAT3, pSTAT3 and β-actin (A) or Raptor, pRaptor and β-actin (C). Representative bar graph showing pSTAT3/STAT3 (B) and pRaptor/Raptor (D) ratios for dalteparin treated villous fragments compared to control. Data are presented as relative means ± SEM. *p<0.05 compared to untreated control (set to 1).

To determine whether the changes in system A and system L amino acid transporter activity after treatment with dalteparin are associated with changes in the JAK/STAT or mTOR pathways we determined the phosphorylation levels of STAT3 and Raptor by Western blot analysis. Incubation of placental villous explants with dalteparin decreased STAT3 phosphorylation under hypoxic conditions (pSTAT3/STAT3, 2.5 IU/ml: 0.58±0.08, p = 0.002, n = 6, table 1), (figure 3A, B). The phosphorylation of Raptor was increased under hypoxic and standard culture conditions although not significantly (figure 3A, B).

### Effect of ASA on Phosphorylation of STAT3 and Raptor at Different Oxygen Levels

**Figure 4:**
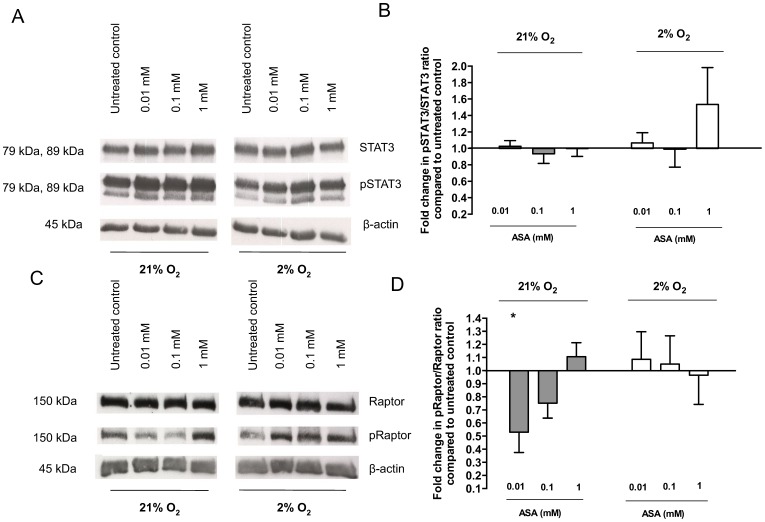
Protein expression of pSTAT3/STAT3 (A, B) and pRaptor/Raptor (C, D) after 2 h of incubation with ASA (0.01 mM, 0.1 mM, 1 mM) at different oxygen levels (21% and 2% O_2_). Representative Western blot of STAT3, pSTAT3 and β-actin (A, C). Representative bars (B, D) show effects on pSTAT3/STAT3 and pRaptor/Raptor ratios compared to untreated control. Data are presented as relative means ± SEM. *p<0.05 compared to untreated control (set to 1).

ASA treatment did not change STAT3 phosphorylation levels as the ratio of pSTAT3/STAT3 after 2 h of incubation with 0.01 mM (1.14±0.09, p = 0.34), 0.1 mM (1.16±0.14, p = 0.5) and 1 mM ASA (1.91±0.21, p = 0.12) under hypoxic conditions, (n = 5, figure 4A, B). Phosphorylation of STAT3 was also not different at 21% O_2_ after a 2 h incubation with different concentrations of ASA.

Under standard culture conditions we observed a significant decrease of Raptor phosphorylation with 0.01 mM ASA (0.66±0.09, p = 0.03, n = 5). ASA at 0.1 mM (0.75±0.11, p = 0.08) and at 1 mM (1.11±0.10, p = 0.36) had no significant effect on phosphorylation of Raptor (n = 6), (figure 4C, D).

### Treatment of Villous Fragments did not Impact LDH Release or hCG Secretion into the Media

**Figure 5:**
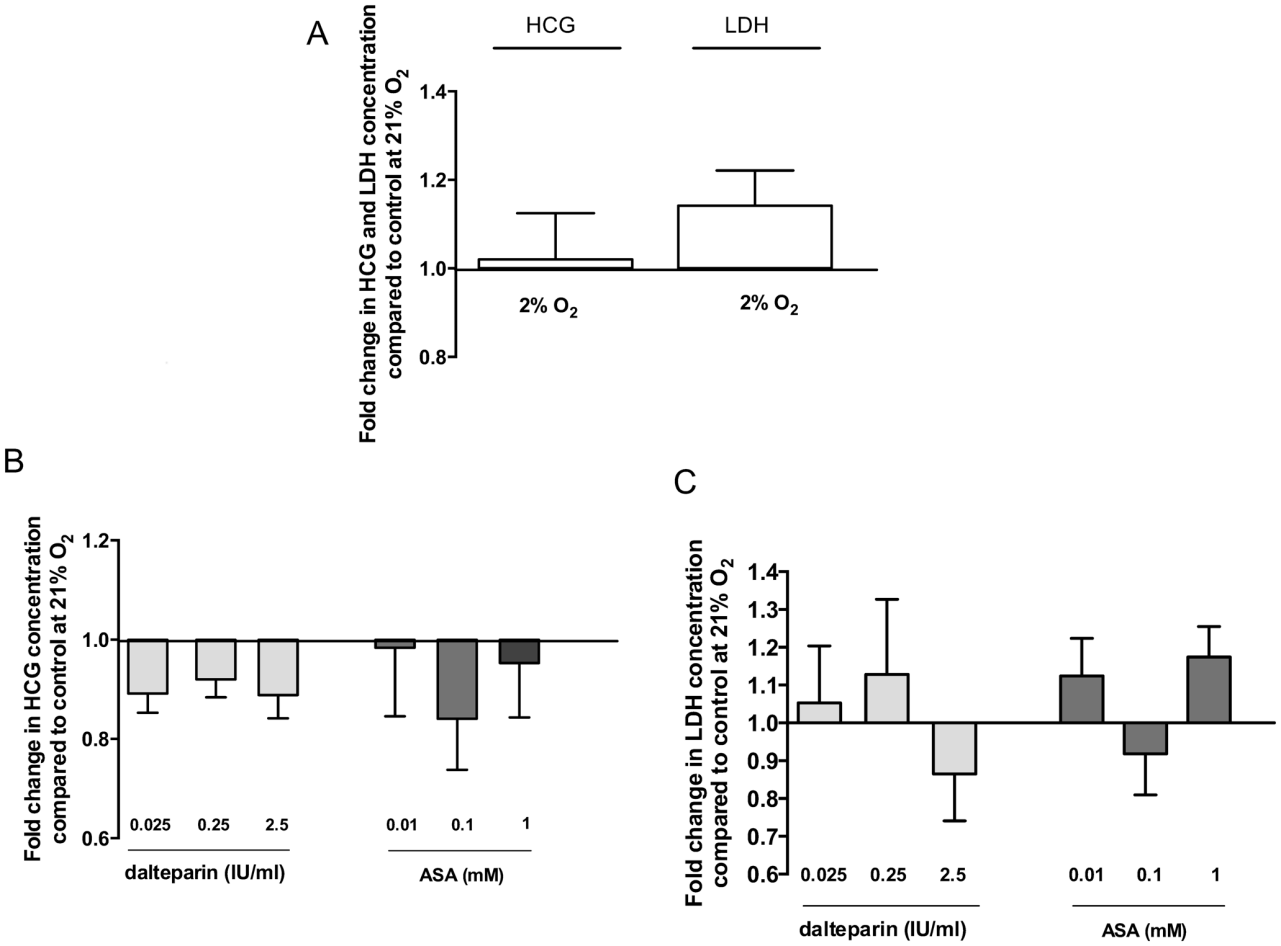
LDH release and secretion of hCG into the culture medium under hypoxic conditions (2% O_2_) compared to 21% O_2_ (A) and after treatment of placental villous explants with dalteparin or ASA at an oxygen level of 21% (B, C). Data are presented as relative means ± SEM. *p<0.05 compared to untreated control (set to 1).

Incubation of villous fragments under different oxygen conditions (figure 5A) or with the different treatment solutions was not associated with a significant change in LDH release or hCG secretion into the media compared to control (figure 5B, C). Therefore, the observed effects on system A and L activity appear unrelated to loss of cell viability and function during incubation.

## Discussion

Our work supports the hypothesis that hypoxia induces placental dysfunction and affects placental nutrient transport. In this study we employed an *ex vivo* placental villous fragment model to explore the hypothesis that hypoxic conditions exert negative effects on placental amino acid transporter activities and that the anticoagulants dalteparin and acetylsalicylic acid (ASA) are capable of neutralizing negative effects of low oxygen conditions on placental amino acid transport. Our data demonstrate that in a hypoxic (2% O_2_) environment the activities of the system A and L amino acid transporters are opposingly affected. We observed a significant decrease in villous explant system A activity and an increase in system L activity in response to low levels of oxygen (2% O_2_) compared to standard culture conditions (21% O_2_). An intermediate oxygen level of 8% O_2_ also enhanced transport of amino acids by system L compared to standard culture conditions. Under hypoxic conditions dalteparin and ASA did not exert a beneficial or rescuing effect on transporter activities. However, under standard culture conditions (21% O_2_) therapeutic levels of dalteparin interacted with third trimester placental villi in a manner that is predicted to have negative effects upon the placental system A and L transport. Similarly, therapeutic or above maximal levels of ASA used in pregnancy appeared to decrease system A activity. While exploring possible signalling mechanisms involved in these changes we found a reduction in STAT3 and a stimulation of Raptor phosphorylation under hypoxic conditions.

Placenta-mediated pregnancy complications are a result of a deficiency in the utero-placental circulation due to thrombosis or impaired trophoblast invasion into the maternal vessels. Placental hypoperfusion and villous hypoxia are observed in preeclampsia and severe IUGR [27], [28], [29]. Substantial confusion concerning oxygen levels for placental tissue culture has arisen during the last decade. Ambient oxygen levels can have marked effects on the actitivity of placental explants. As stated by Miller et al. and Burton et al. 3% or less oxygen is considered hypoxia for term placenta [30], [31]. Importantly, we found a reduction in system A and an increase in system L transport activity that was dependent on O_2_ concentration. We show that acute hypoxia (2% O_2_) for 2 h leads to a 27% reduced activity of the placental system A amino acid transporter compared to standard culture conditions. The low pO_2_ levels (2% O_2_) used are similar to O_2_ levels observed in fetuses with severe IUGR [29]. Our data are in line with a previous study by Nelson *et al*. in cytotrophoblasts that after 24 h of culture found a 82% reduction in transport activity at 1% O_2_ and a 37% reduction at 3% O_2_ compared with standard conditions (20% O_2_) in cultured term human trophoblasts [32]. Our finding of increased system L transporter activity by 42% at 2% O_2_ is novel. In addition, we choose an intermediate concentration of 8% O_2_ and observed no differences in system A activity but again a significant increase in system L transport activity. Decreased placental system A and L activities have been reported in pregnancies complicated by IUGR [18], [33] but no studies on placental system L activity have been performed so far to elucidate the effect of reduced oxygen conditions. On the basis of our results, we speculate that hypoxia *in vivo* may diminish selected amino acid transporter activities and conversely that higher transport activity of e.g. the system L transporter might represent an adaptive mechanism to compensate for the reduction in other transport systems under altered placental oxygen conditions. This mechanism might explain the different findings of lower fetal blood levels of amino acids transported by system A in IUGR pregnancies compared to normal levels in preeclamptic pregnancies [34], [35], [36].

Several trials have explored the potential effect of prophylactic LMWHs or ASA in improving pregnancy outcome in women with thrombophilia or previous pregnancies that have been complicated by miscarriage, IUGR, preeclampsia, placental abruption, or sudden intrauterine death. To date only ASA started before 16 weeks of pregnancy has been proven to have beneficial effects and reduces the risk for perinatal death, preeclampsia and IUGR [7], [37], [38]. Despite increasing use in pregnancy the interaction between placental nutrient transfer as a feature of placental function and these anticoagulants has not been studied. The second goal of our study was therefore to explore the effect of LMWHs and ASA on placental amino acid transport under low oxygen conditions that simulate placental pathology. Concentrations of the agents chosen for this study reflect therapeutic and supra-therapeutic plasma levels of the respective substances during treatment *in vivo* according to previous studies [23]. A concentration of 1 mM ASA (∼150 mg/d) decreased the activity of system A at 21% O_2_ and 2% O_2_, respectively, whereas the activity of system L decreased only at 2% O_2_. Treatment of primary villous fragments for 2 h with different concentrations of dalteparin did not affect system A or L activity under hypoxic conditions. However, dalteparin decreased system A activity by 22% and system L transport activity by 31% at 21% O_2_.

To our knowledge this is the first study to explore the effect of the anticoagulants dalteparin and ASA on placental system A and L transport. Kinetic studies using a model of isolated perfused cotyledons taken from placentae of aspirin-treated pregnancies showed that L-arginine is transported with a significantly higher affinity, but with a lower capacity than in the non-treated group [39]. The latter finding suggests that ASA would facilitate the uptake of the nitric oxide precursor only at very low arginine concentrations. A significant decrease in histidine transport has also been reported after aspirin treatment in rat intestine [20]. In the few studies available, that explored the effects of hormones or drugs on placental amino acid transport, it has been reported that system A activity was stimulated by insulin, dexamethasone and glucagon in cultured human trophoblast cells [40], [41]. Our own data and others also showed that the adipokine leptin increases placental system A activity by activating the JAK-STAT signalling cascade [17], [21]. Therefore, drug treatment has the capability to modify placental function, e.g. amino acid transport. To further understand the possible mechanisms we focused on two well-studied signalling pathways- the mTOR and JAK/STAT cascade and determined the phosphorylation of Raptor (mTORC1) and STAT3 at the protein level. Our results show that at low oxygen concentrations the phosphorylation state of STAT3 decreased significantly and Raptor, which is part of the mTORC1 complex, increased significantly. Roos *et al.* showed that treatment of placental villous fragments for 4 h with the mTOR inhibitor rapamycin (100 nM), completely abandoned system L activity, whereas system A activity did not change significantly [42]. Hypoxia (1.5% O_2_) rapidly and reversibly triggers hypophosphorylation of mTOR and its effectors in HEK293 cells [42], what is in contrast to our findings of an increased phosphorylation at 2% O_2_ in placental villous fragments. Under hypoxic conditions the expression of STAT3 in vascular smooth muscle cells was reduced [43], which is consistent with our findings. We speculate that there is a balance between both pathways and that this balance is altered under hypoxic conditions such that the mTOR pathway is up-regulated and STAT3 signalling is down-regulated. This in turn could lead to a stimulation of system L activity and an inhibition of system A activity. To further evaluate whether system L activity is indeed regulated by different oxygen levels through the mTORC1 complex, the inhibition of mTORC1 by rapamycin would provide additional insights on this pathway.

Our data on LMWHs and ASA effects on mTOR and STAT3 signalling currently do not strongly support our hypothesis of a regulation of these pathways. Although we observed a reduction in STAT3 phosphorylation under hypoxic conditions in the presence of dalteparin, this effect was only found at the highest concentration.

In summary, we observed an oxygen dependent effect on amino acid transport systems A and L in placental villous explants. Additionally, we found that treatment with ASA or dalteparin negatively affected the activity of both transporter systems under standard culture conditions. However, under hypoxic conditions only ASA further reduced the activity of system A and L. The STAT3 and mTOR pathway appear to be involved in the oxygen dependent regulation either directly or indirectly. In conclusion, dalteparin and ASA do not neutralize the negative effect of hypoxia and therefore pathologic conditions on placental system A and L transport. Our data support the demand for further investigations of ASA and LMWH effects on placental function, the avoidance of blind use and use beyond a certain stage of pregnancy, e.g. third trimester.
